# Anatomical Features of the Sacroiliac Joint and Machine Learning-Based Classification of Disease Types

**DOI:** 10.3390/diagnostics16050687

**Published:** 2026-02-26

**Authors:** Rabia Koca, Fatih Ateş, Yavuz Bahadır Koca, Zeliha Fazlıoğulları, Mehmet Sedat Durmaz

**Affiliations:** 1 Department of Physical Therapy and Rehabilitation, Faculty of Health Sciences, Afyonkarahisar Health Sciences University, Afyonkarahisar 03030, Türkiye; 2 Department of Radiology, Konya City Hospital, Health Ministry of Turkish Republic, Konya 42020, Türkiye; fatih_ates81@hotmail.com; 3 Department of Electrical Engineering, Faculty of Engineering, Afyon Kocatepe University, Afyonkarahisar 03200, Türkiye; ybkoca@gmail.com; 4 Department of Anatomy, Selcuk University, Konya 42130, Türkiye; z_topal@yahoo.com; 5 Department of Radiology, Selcuk University, Konya 42130, Türkiye; dr.msdurmaz@gmail.com

**Keywords:** sacroiliac joint, morphology, morphometry, inflammation, degeneration, artificial intelligence, magnetic resonance imaging

## Abstract

**Background and Objectives:** Understanding the structural differences in the sacroiliac joint (SIJ) is essential for distinguishing inflammatory from degenerative disorders. This study aimed to evaluate disease-related morphological patterns and morphometric characteristics of the sacroiliac joint. Additionally, machine learning models were applied to classify inflammatory, degenerative, and control groups based on the morphological and morphometric characteristics of the sacroiliac joint. **Materials and Methods:** This retrospective study included Magnetic Resonance Imaging (MRI) images of 209 individuals (a total of 418 sacroiliac joints) between the ages of 18 and 75. Participants’ age, sex, disease-related sacroiliac joint morphological features (joint surface type), erosion, sclerosis and inflammation in the joint were determined. Right/left joint space and right/left joint length were measured. According to these anatomical features, machine learning models and a deep neural network were used to classify joints as control, inflammatory, or degenerative. Stratified 5-fold cross-validation was used. Results were reported as mean ± SD with macro averaged precision, recall, and F1-score. **Results:** The degenerative group was significantly higher than the other groups in terms of mean age (*p* = 0.001). Both right and left sacroiliac joint spaces were significantly narrower in the inflammatory and degenerative groups than in controls (right SIJ space: *p* = 0.002; left SIJ space: *p* = 0.001). Erosion was significantly more frequent in pathological groups (*p* = 0.001). Although the iliosacral complex was the most common joint type in all groups, no significant difference was observed between the disease groups (right, *p* = 0.852; left, *p* = 0.935). In classification, SVM (RBF) and XGBoost achieved the highest accuracy (both: 0.9518 ± 0.0380 and 0.9518 ± 0.0436, respectively) and macro-F1 (0.9509 ± 0.0387 and 0.9506 ± 0.0443). **Conclusions:** Disease-related morphological and morphometric changes in the sacroiliac joint can be reliably assessed with MRI. These features can then be used in machine learning models to differentiate between inflammatory and degenerative joint disorders. Carefully examining these anatomical features plays a key role in reaching an accurate diagnosis. Machine learning supports this process by helping to interpret the findings in a more consistent and objective way.

## 1. Introduction

The sacroiliac joint (SIJ), located between the sacrum and the ilium, plays a critical role in mechanical stability and load transfer from the spine to the lower extremities [1,2]. Due to its limited range of motion (~1 mm), even minor structural alterations in the SIJ can trigger clinical symptoms such as low back pain [3,4].

The morphology and morphometry of the SIJ, including the shape and structure of joint surfaces, show significant inter-individual variability. Relationships with surrounding tissues, physiological and pathological conditions can also affect this diversity [5,6]. Inflammatory and degenerative conditions lead to distinct structural changes in the SIJ, potentially resulting in disease-related morphological joint surface [7,8]. Inflammatory changes may result in erosion, sclerosis, or bone marrow edema, and can progress to joint surface fusion (ankylosis) in severe cases [9,10].

Degenerative problems are often associated with aging, microtraumas and biomechanical disorders [11,12,13,14,15]. They are characterized by changes such as cartilage loss, subchondral sclerosis, and osteophyte formation [16,17]. These differences can lead to an asymmetric joint structure and a more complex clinical condition. Accurate evaluation of these structural alterations requires high-resolution imaging and objective morphometric analysis.

Recent progress in quantitative imaging techniques has facilitated the objective assessment of anatomical structures in clinical research [18]. Machine learning (ML) methods have been employed to support morphological evaluations by reducing observer variability. ML has proven effective in radiology and medical image analysis, particularly in classifying morphological changes and performing tasks such as sex estimation. These methods have also been increasingly applied to the sacroiliac joint and other anatomical regions [19,20,21]. By reducing subjectivity in MRI interpretation, learning algorithms improve diagnostic accuracy and support clinical decision-making, as demonstrated in studies detecting sacroiliitis from imaging data [22,23,24].

In the literature, inflammatory and degenerative sacroiliac joint diseases are generally examined separately, or the focus is on descriptive anatomical variations [1,5,6,25,26]. However, this study allows a direct comparison of disease-related structural changes by combining inflammatory, degenerative, and control groups within a single analytical framework. Recent studies have mostly used machine learning or deep learning methods to detect inflammatory sacroiliitis [27,28]. Many of these studies are based on radiomic or black box modeling approaches with limited anatomical interpretability [29,30].

Therefore, a study evaluating inflammatory and degenerative sacroiliac joint diseases together and comparing them with a control group was needed. The primary motivation for this study has been the classification of sacroiliac joint diseases based on disease-related anatomical joint surface types of the sacroiliac joint and morphometric parameters on MRI, and their objective and comparative evaluation using machine learning.

This study retrospectively examined morphological and morphometric changes associated with inflammatory and degenerative pathologies in the SIJ. Anatomical changes in the SIJ are evaluated with morphological and morphometric parameters obtained from MRI data. In the literature, diseases are usually examined independently [8,10,16], but this study examines both problems together and reveals the pathological changes in the SIJ comparatively. The anatomical findings obtained are systematically compared with those of the control group. An additional aim is to assess whether machine learning-based models can accurately classify joints into inflammatory, degenerative, or control categories based on morphometric input. Such tools may complement conventional evaluation by providing consistent and reproducible classification of structural joint changes.

This study offers two main contributions.

1. It aims to understand better the morphological and morphometric differences between inflammatory and degenerative diseases of the sacroiliac joint.
2. It seeks to demonstrate the applicability of machine learning methods to radiological data and their integration into clinical decision support systems. Therefore, this study bridges the gap between descriptive anatomical investigations and data-driven diagnostic support for sacroiliac joint disorders by combining comparative pathology analysis with interpretable machine learning.

## 2. Materials and Methods

This retrospective, cross-sectional study investigated structural variations in the sacroiliac joint (SIJ) using MRI-based morphological and morphometric assessments. MRI scans were evaluated to distinguish inflammatory, degenerative, and control groups according to predefined radiological criteria. Morphological and morphometric variables were extracted from MRI. Statistical analyses and supervised machine learning algorithms were used to identify group-specific patterns. Model performance was assessed using stratified 5-fold cross-validation.

### 2.1. Study Design and Ethical Approval

**Figure 1:**
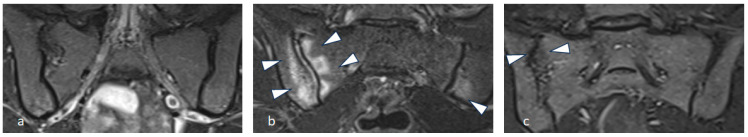
Representative MRI images of sacroiliac joints: (**a**) Control SIJ; (**b**) Inflammatory SIJ; (**c**) Degenerative SIJ.

This single-center, retrospective study was conducted using anonymized pelvic MRI scans obtained from the Radiology Department of Selçuk University. The study complied with the principles of the Declaration of Helsinki. All MRI data were used exclusively for research purposes, with no clinical interventions performed. Representative MRI examples from the control, inflammatory, and degenerative groups are provided in Figure 1.

### 2.2. Study Population and Dataset Construction

A total of 209 adult individuals aged 18 to 75 years were included. Participants were categorized into three groups based on radiological assessment: inflammatory (*n* = 69), degenerative (*n* = 64), and control (*n* = 76). Group assignment was primarily based on MRI findings, and available clinical information was reviewed as supportive context. For the inflammatory group, inclusion required MRI findings consistent with sacroiliitis according to the Assessment of SpondyloArthritis international Society (ASAS) classification criteria, including bone marrow edema, subchondral sclerosis, and/or erosions, with or without joint space narrowing. Where possible, clinical information (e.g., back pain history, laboratory markers) was reviewed, but primary classification was based on imaging in all cases.

The degenerative group was defined by cartilage loss, osteophyte formation, and sclerosis in the absence of any active inflammatory findings. The control group consisted of individuals with normal SIJ morphology and no radiological evidence of either degeneration or inflammation. Because MRI in the control group was performed for non-specific complaints, subclinical SIJ pathology cannot be fully excluded. Group classification was based on imaging features reviewed by radiologists, with discrepancies resolved by consensus. Table 1 presents the distribution of participants by age and sex for SIJ.

➢ **Inflammatory group:** Characterized by bone marrow edema, subchondral sclerosis, erosions, or joint space narrowing indicative of sacroiliitis.
➢ **Degenerative group:** Identified by cartilage loss, osteophyte formation, and sclerosis, in the absence of active inflammation.
➢ **Control group:** Displayed normal joint morphology without signs of degeneration or inflammation.

The dataset included demographic variables (age, sex), bilateral morphometric measurements (SIJ space and anteroposterior length), categorical joint types (left/right), and binary radiological features (erosion, sclerosis, and inflammation), which were recorded in a structured spreadsheet for statistical analysis and model training.

**Inclusion criteria: **

• Age between 18 and 75 years;
• Bilateral sacroiliac joint MRI of diagnostic quality;
• Adequate visibility of joint margins and adjacent bony structures.

**Exclusion criteria:**

• History of pelvic trauma or sacroiliac joint surgery;
• Presence of congenital anomalies or neoplastic lesions in the pelvic region;
• Poor-quality MRI scans due to motion artifacts or insufficient resolution.

**Figure 2:**
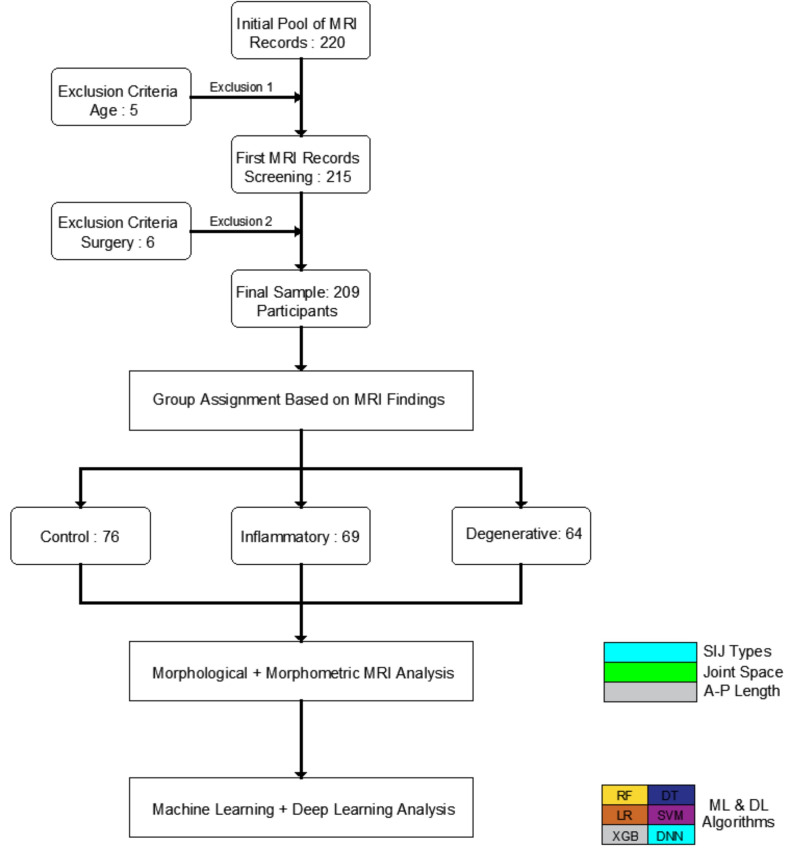
Flow diagram of the study design.

Demographic variables were recorded and used in the analysis of SIJ morphology and morphometry. The overall study workflow is illustrated in Figure 2.

### 2.3. MRI Acquisition and Imaging Protocol

All SIJ MRI scans were acquired in 2024 using a 1.5 Tesla MRI system (Magnetom Aera, Siemens Healthcare, Erlangen, Germany) and standardized pelvic imaging protocols. All examinations were performed on the same scanner using the same protocol. Participants were scanned in the supine position. Coronal and axial sequences were acquired to evaluate joint morphology and potential pathological features with high spatial resolution. The following MRI sequences were used:

Coronal T2-weighted short tau inversion recovery (STIR): TR/TE = 4080/54 ms;

Coronal T1-weighted: TR/TE = 600/20 ms;

Coronal T1-weighted fat-suppressed: TR/TE = 556/12 ms;

Axial proton density fat-suppressed: TR/TE = 2440/29 ms.

All scans were obtained at the level of the second sacral vertebra (S2), corresponding to the midpoint of the sacroiliac joint. Slice thickness was maintained at 3–4 mm, with a field of view (FOV) of 240–300 mm and appropriate matrix dimensions to ensure diagnostic quality. Images were assessed using Picture Archiving and Communication System (PACS) software. Two radiologists with over five years of musculoskeletal imaging experience independently reviewed the images while being blinded to clinical information and study grouping. Discrepancies in assessment were resolved through consensus review.

### 2.4. Morphological and Morphometric Analysis

**Figure 3:**
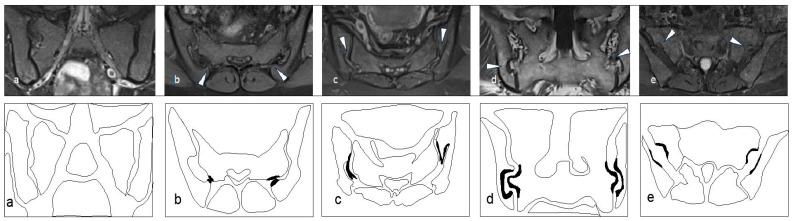
Representative MRI images showing anatomical variations in the sacroiliac joint (Arrowheads point to SIJ): (**a**) Normal SIJ with no variation; (**b**) Bilateral accessory type; (**c**) Bipartite type; (**d**) Bilateral iliosacral complex type; (**e**) Left dominant semicircular defects type.

Morphological evaluation included visual classification of joint types and pathological features based on coronal and axial MRI images. Morphometric evaluation was performed by quantitatively measuring the SIJ space and the anteroposterior length. Each SIJ was categorized into one of the following types: normal, accessory, bipartite, iliosacral complex, or semicircular defects [6,8]. This classification was made based on the morphological appearance of the joint surface on coronal T2-weighted MRI images. In this classification, the term “normal” denotes the standard joint surface appearance without a defined anatomical variant. Group labels (control/inflammatory/degenerative) were assigned independently based on radiological criteria, and joint-surface types were compared across these groups. Both right and left SIJs were evaluated, and distribution of types was compared between groups (Figure 3).

In addition to morphological evaluation, two morphometric parameters were measured:

• SIJ space: the distance between sacral and iliac joint surfaces [20];
• Anteroposterior joint length: the distance from the anterior to the posterior aspect of the joint [20].

**Figure 4:**
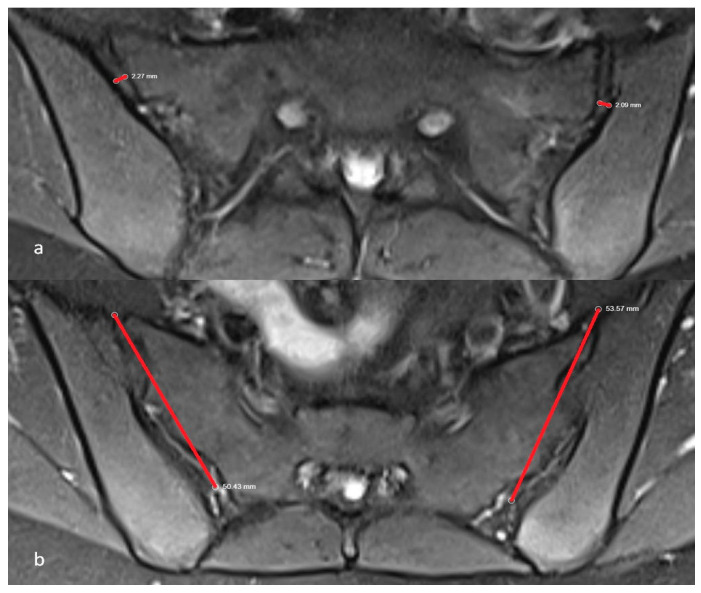
Axial proton density fat-suppressed MRI images at the level of the S2 vertebra: (**a**) The SIJ space; (**b**) The anteroposterior lengths of the SIJ.

Both measurements were made at the level of the S2 vertebra using axial proton density fat-suppressed sequences, which provided standardized imaging for consistent anatomical assessment. Joint space was evaluated at the corpus level, while anteroposterior length was measured at the midsection of the vertebral body (Figure 4a,b). These morphometric criteria were selected due to their diagnostic relevance in distinguishing inflammatory and degenerative joint changes. In addition, MRI samples from the diagnostic groups and detailed information on data preprocessing are provided in the Supplementary Figures S1–S5.

### 2.5. Data Preprocessing and Feature Engineering

Before model training, some preprocessing steps were applied to ensure data quality and handle feature scaling and categorical encoding. First, all continuous variables, including SIJ space and A–P length, were standardized using StandardScaler (z-score normalization). This step reduces the influence of differing numeric ranges and is particularly important for models that are sensitive to feature scaling (e.g., SVM). To prevent data leakage, scaling parameters were learned only from the training data within each cross-validation fold and then applied to the corresponding test fold.

Categorical variables (sex, joint types, and binary radiological features) were transformed using one-hot encoding to enable their use in the models. This preprocessing pipeline generated a structured feature representation suitable for statistical analysis and model training.

### 2.6. Machine Learning and Deep Learning

To enhance diagnostic differentiation between the inflammatory, degenerative, and control groups, supervised machine learning (ML) algorithms were applied to the morphological and morphometric features derived from MRI. The dataset included the following input variables: age, sex, joint space (right/left), anteroposterior joint length (right/left), presence of erosion, sclerosis, and inflammation, and anatomical joint type.

**Figure 5:**
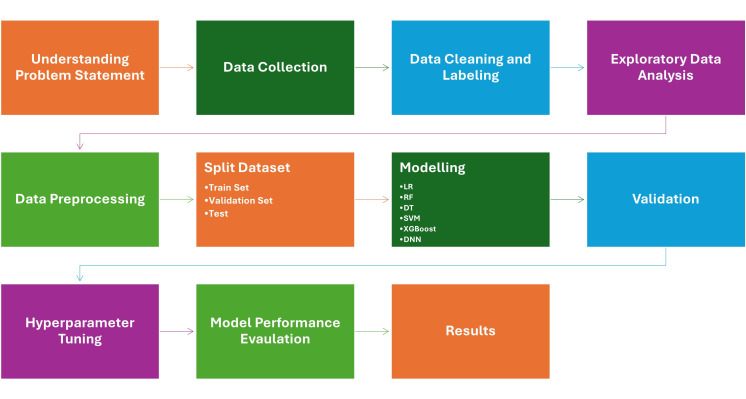
Workflow diagram illustrating the sacroiliac joint (SIJ) classification pipeline, including MRI acquisition, feature extraction, group assignment, and machine learning-based diagnostic modeling.

In this study, five supervised ML classifiers were developed and compared. These are, logistic regression (LR), RBF kernel support vector machine (SVM), decision tree (DT), random forest (RF), and XGBoost. Moreover, a deep neural network (DNN) model was constructed to model non-linear relationships and feature interactions that may not be captured by linear classifiers. Model performance was evaluated using the following metrics: accuracy, precision, recall, F1-score, and area under the receiver operating characteristic curve (AUC) [31]. For DL, a feedforward multilayer perceptron (scikit-learn MLPClassifier) was trained using the same tabular input features. The architecture consisted of three hidden layers (128–64–32) with ReLU activation and the Adam optimizer and L2 regularization (α = 1 × 10^−4^, learning rate = 0.001, random_state = 42). Training was performed for up to 200 epochs with early stopping based on validation log loss (patience = 20, min_delta = 1 × 10^−6^), and the best-performing model was retained. A summary of the ML analysis workflow is illustrated in Figure 5.

### 2.7. Statistical Analysis

All statistical analyses were performed using IBM SPSS Statistics (version 26.0, IBM Corp., Armonk, NY, USA). The distribution of numerical variables was assessed using the Shapiro–Wilk test. Data with normal distribution were presented as mean ± standard deviation (SD), while categorical variables were expressed as frequencies and percentages. For comparisons between the three groups (inflammatory, degenerative, control), the following statistical tests were used:

One-way analysis of variance (ANOVA) for normally distributed continuous variables, followed by Tukey’s post hoc test where appropriate.

Kruskal–Wallis test was applied when the data were not normally distributed.

Chi-square (χ^2^) test was used to evaluate differences in categorical variables such as anatomical joint type.

The level of statistical significance was set at *p* < 0.05. All statistical tests were two-tailed. Inter-observer agreement between the two radiologists was evaluated using Cohen’s kappa (κ) coefficient for categorical variables, and intraclass correlation coefficient (ICC) for continuous measurements.

## 3. Results

The results are structured around four main components: demographic and morphometric characteristics, pathological features observed in MRI assessments, disease-related anatomical joint surface types of the sacroiliac joint, and the classification performance of machine learning and deep learning models. Statistical analyses were performed to compare the groups, while supervised learning techniques were used to evaluate the diagnostic value of the anatomical parameters. All findings are reported with corresponding *p*-values and model performance metrics.

### 3.1. Demographic and Morphometric Findings

A total of 209 individuals were included in the study. The participants were categorized into three groups: control (*n* = 76), inflammatory (*n* = 69), and degenerative (*n* = 64). The mean age of the degenerative group (53.23 ± 8.27 years) was significantly higher than that of the control (36.80 ± 11.02 years) and inflammatory (40.96 ± 13.95 years) groups (*p* < 0.05).

**Figure 6:**
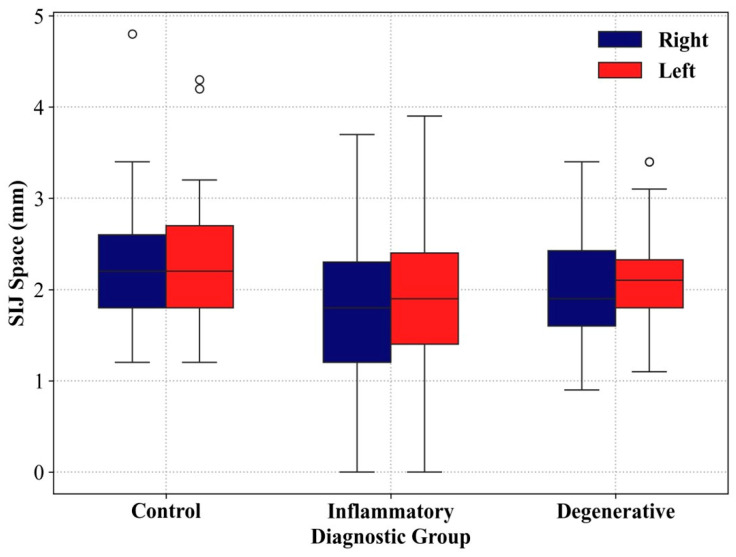
Right and left sacroiliac joint space measurements by diagnostic group.

Morphometric evaluation showed that both right and left sacroiliac joint spaces were significantly narrower in the inflammatory and degenerative groups compared to the control group (*p* < 0.05). Figure 6 shows boxplots of right and left SIJ space measurements across diagnostic groups. However, there was no statistically significant difference between the groups in terms of right and left anteroposterior joint lengths (*p* > 0.05). These results indicate that joint space narrowing may be a more sensitive parameter than joint length in distinguishing between pathological and non-pathological conditions (Table 2).

### 3.2. Pathological Features

In addition to morphometric findings, pathological alterations such as erosion, sclerosis, and inflammation were assessed across the three groups. Erosion was significantly more frequent in the inflammatory and degenerative groups compared to the control group (*p* < 0.05). While only 12 individuals in the control group exhibited erosion, this number increased to 56 in the inflammatory group and 63 in the degenerative group.

Similarly, sclerosis was observed more frequently in pathological groups, with a significant increase compared to controls (*p* < 0.05). Inflammation, as expected, was almost exclusively present in the inflammatory group. Among these, 19 individuals exhibited active inflammation, 18 had chronic inflammation, and 30 showed a combination of both. These findings confirm that inflammatory processes produce distinct pathological patterns on imaging, which are rarely observed in degenerative cases.

Regarding joint type, the iliosacral complex was the most commonly observed anatomical variant across all groups. However, no statistically significant difference was found in the distribution of joint types among the three groups (*p* > 0.05), suggesting that joint type alone may not be a reliable marker for differentiating pathological conditions. These results are detailed in Table 3, which summarizes the frequency and statistical significance of pathological findings and joint type distributions.

### 3.3. Disease-Related Anatomical Joint Surface Types

Disease-related anatomical joint surface types of the sacroiliac joint were assessed based on previously defined morphological types, including normal, accessory joint, bipartite, iliosacral complex, and semicircular defect. Each sacroiliac joint was classified independently on the right and left sides. Among all evaluated joints, the iliosacral complex was the most frequently observed variant in all three groups.

Although the iliosacral complex type appeared slightly more often in the degenerative group, statistical analysis revealed no significant difference in joint type distribution among the control, inflammatory, and degenerative groups for either the right (*p* = 0.852) or left (*p* = 0.935) side. These findings suggest that anatomical joint variations are relatively consistent across clinical conditions and may not serve as reliable indicators for distinguishing inflammatory or degenerative changes. This distribution is summarized in Table 3, which includes the frequency and statistical significance of disease-related anatomical joint surface types.

### 3.4. Modeling Performances

Five different ML algorithms and one DNN models were used to evaluate the differentiation of sacroiliac joint pathologies (inflammatory, degenerative, and control). LR, RBF-kernel SVM, DT, RF, and XGBoost models were tested along with a DNN model. All models were trained using the same feature set. Comparison was performed using stratified 5-fold cross-validation. Z-score standardization was applied to continuous variables. Additionally, one-hot coding was applied to categorical variables within each fold to prevent potential data leakage. Performance results were reported as mean ± standard deviation calculated from the outputs of each fold. Macro-averaged precision, recall, and F1-score were used to give equal weight to each class in the three-class structure.

**Figure 7:**
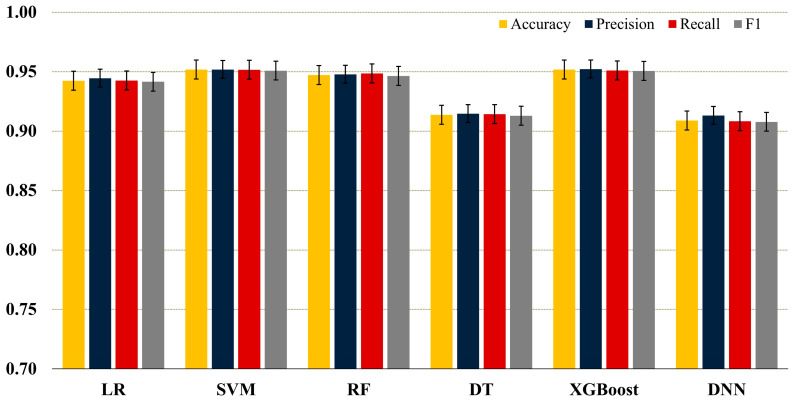
Performance of ML and DNN models for SIJ pathology classification.

Among the ML models, the highest accuracy values were obtained with SVM and XGBoost. The macro-F1 scores of these two models were also found to be similarly high (SVM: 0.9509 ± 0.0387, XGBoost: 0.9506 ± 0.0443). RF and LR models also achieved results close to the other two algorithms (RF accuracy: 0.9472 ± 0.0242, LR accuracy: 0.9424 ± 0.0250). However, the DT model showed relatively lower performance (accuracy: 0.9137 ± 0.0293). Macro-average metrics for all models are presented in Table 4, and a comparative performance visualization is given in Figure 7.

A DNN was also applied to the dataset with ML models. This analysis aimed to here was to model the nonlinear relationships and higher-level interactions between tabular MRI-based features and observe the outputs. The DNN was trained and evaluated with stratified 5-fold cross-validation. Within each outer fold, the training section for early stopping was further split into 80%/20% inner training/validation. Early stopping validation was performed according to the log-loss value. To prevent data leakage, standardization of continuous variables and one-hot encoding of categorical variables were learned only on the inner training data. Then, they were applied to the relevant validation and testing sections.

The network structure was established as a multilayer perceptron (MLP) consisting of three hidden layers (128–64–32) with ReLU activation. A three-node softmax output layer was used for multi-class prediction. Optimization was performed with Adam (initial learning rate = 0.001) and L2 regularization was applied (α = 1 × 10^−^^4^). Training was conducted for a maximum of 200 epochs. Early stopping was triggered when no improvement was observed in validation loss over 20 consecutive epochs.

Across cross-validation folds, DNN achieved a macro-mean accuracy of 0.9089 ± 0.0572. Macro-precision was found to be 0.9132 ± 0.0530, macro-recall (sensitivity) 0.9083 ± 0.0583, and macro-F1 0.9078 ± 0.0575 (Table 4). Although DNN performance was somewhat lower than that of the highest-performing classifiers, it showed consistent behavior in multi-class discrimination. It provided a nonlinear basis for comparison in addition to ML models. In Figure 7, the bars show the average of the macro-averaged accuracy, precision, recall, and F1 values obtained with stratified 5-fold cross-validation. The error bars show the ±SD value between the folds.

**Figure 8:**
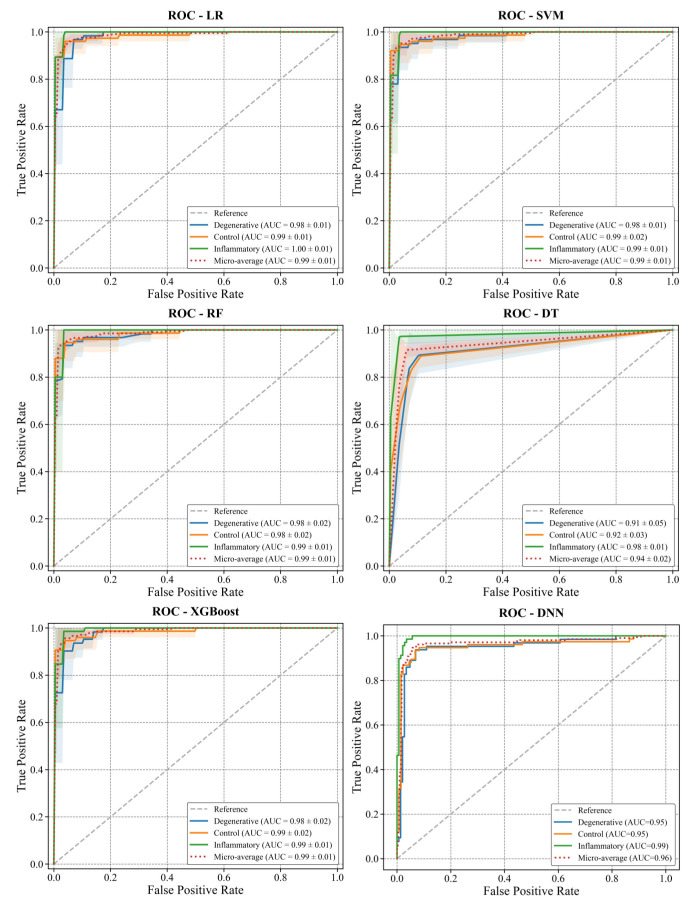
Cross-validated ROC curves of the models for SIJ classification.

**Figure 9:**
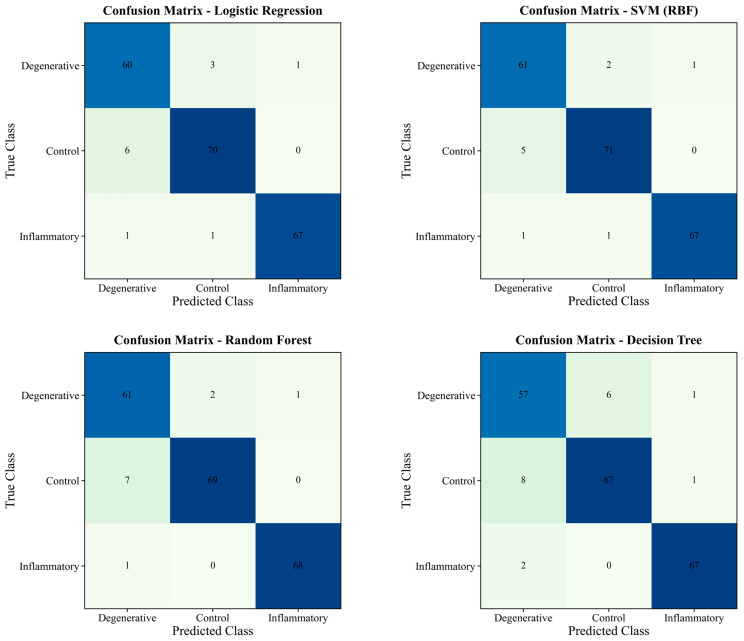

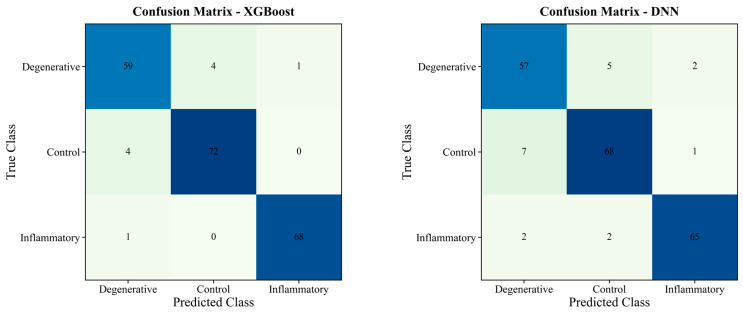
Confusion matrices of the models for SIJ classification.

ROC curves and confusion matrices were calculated the cross-validated performance metrics. In this context, ROC curves obtained from cross-validation probability outputs are presented in Figure 8. Using the relevant confusion matrices, inter-class separation and error patterns are visually shown in Figure 9. When these findings are considered together, SVM and the ensemble-based models (RF and XGBoost) showed consistently strong discrimination between inflammatory, degenerative, and control groups with the current set of variables. Figure 8 shows the micro-average curve along with the one-vs-rest ROC curves for degenerative, control, and inflammatory classes for each model. The curves were constructed from stratified 5-fold cross-validation probability outputs. AUC values are reported as mean ± SD across the folds. The diagonal dashed line indicates the reference (random) level. Matrices summarizing class-based error patterns for three groups, derived from cross-validation estimates, are shown in Figure 9. Values in the cells represent the sample size.

**Figure 10:**
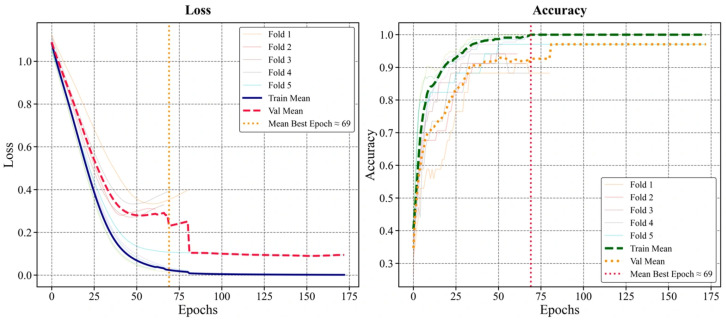
Learning curves of the DNN model under stratified 5-fold cross-validation.

The learning behavior of the DNN was investigated using a stratified 5-fold cross-validation, similar to that in ML models. In each outer fold, the training data for early stopping was further subdivided into inner training/validation subsets. Model parameters were selected based on the epoch with the lowest validation log-loss. Fold-based learning curves showed that both the training and validation losses decreased consistently as the epochs progressed, while the accuracy values stabilized and increased. No significant divergence was observed between the training and validation curves, indicating stable convergence. The average number of best selected epochs across the folds was approximately 69. After this point, performance gains were limited. These learning dynamics are summarized in Figure 10, showing the average training/validation curves for each fold superimposed. Furthermore, the macro-mean specificity (OvR) for DNN was calculated as 0.9547 ± 0.0287, and the macro-mean OvR AUC as 0.9828 ± 0.0133.

## 4. Discussion

Inflammatory and degenerative changes in SIJ occur through different mechanisms. Goldring (2016) stated that narrowing or widening of the joint space may be observed in areas where inflammation is intense [32]. McGonagle et al. (2014) and Ziegeler et al. (2023) showed that erosions and bone marrow edema on the joint surface disrupt the joint structure and lead to morphological variations [9,33]. It has been emphasized in previous studies that chronic inflammation causes permanent disorders and sclerotic changes. Carotti et al. (2024) reported that severe inflammation leads to the fusion of joint surfaces (ankylosis), significantly changing the joint’s morphological type and movement capacity [10].

On the other hand, degenerative processes are generally associated with aging, and deterioration of joint biomechanics or repetitive microtraumas can accelerate this process [8]. As a result of degenerative changes, cartilage loss is observed in SIJ, and subchondral sclerosis, osteophyte formation and narrowing of the joint space are common findings [16]. This process disrupts the smoothness of the joint surfaces, causing morphological asymmetry and, in advanced stages, leading to osteophyte formation at the joint edges. These changes affect the joint type, making the clinical picture more complex [17].

It is important to accurately distinguish between inflammatory and degenerative processes in determining the most appropriate treatment method for patients. Because therapeutic approaches for inflammatory and degenerative problems differ significantly [34]. Unlike previous studies, inflammatory and degenerative disease groups were compared with the control group in this study. The findings support that the mean age of the degenerative group was significantly higher than that of the other groups and that degeneration increases with aging. In addition to the findings reported in the literature, the right and left SIJ spaces were found to be significantly narrowed in the inflammatory and degenerative groups (*p* < 0.05). In addition, in four patients in the inflammatory group, complete closure of the joint space was observed depending on the severity of inflammation. These results confirm the changes previously reported in the literature. In other words, they show again that inflammatory and degenerative processes affect joint morphology with different mechanisms. Differentiating inflammatory and degenerative changes in SIJ imaging poses a significant challenge due to the similar radiological features of both conditions. Findings such as erosion, sclerosis, and bone marrow edema are common in inflammatory and degenerative processes and complicate the diagnostic process. Carotti et al. (2024) explained in their study that clinical conditions such as osteitis condensans ilii can mimic inflammatory sacroiliitis and make diagnosis and treatment decisions difficult [10]. Malghem et al. (2007) revealed in their study that it can be similar to inflammatory lesions of mechanical stress and ligament ossifications [35].

Due to these similarities, careful evaluation of structural lesions is important for correct diagnosis. Gensler et al. (2024) stated that bone marrow edema can occur in different pathologies [36]. Therefore, specific imaging methods are needed to distinguish inflammatory and degenerative changes. Kepp et al. (2021) emphasized the importance of de-tailed interpretation to prevent misclassifications [37]. They also suggested the use of advanced analytical methods. Golovach et al. (2021) stated that limited access to imaging methods, slow progress of radiological developments, and lack of expertise in the interpretation process are factors that make it difficult to make a correct diagnosis [38].

The spreading characteristics of inflammatory and degenerative changes may be different. Malghem et al. (2007) stated that inflammatory changes are generally more extensive and widespread [35]. However, they stated that degenerative changes are more localized and compact. Therefore, they revealed the importance of comprehensive evaluation in the diagnostic process. In this study, joint erosion and sclerosis were found to be significantly increased in both inflammatory and degenerative disease groups compared to the control group. Active and chronic inflammation was significantly increased only in inflammatory joint diseases. In addition, osteitis condensans was detected in one patient in the inflammatory group. This situation shows that it is difficult to distinguish inflammatory and degenerative changes. The findings in the study are consistent with the studies in the literature. This supports the need for more sensitive evaluation criteria to distinguish inflammatory and degenerative processes.

Umay and Korkmaz (2019) found six types of variations in the SIJ [25]. They stated that the most common type was the iliosacral complex (7.7%). Vereecke etal. (2023) found eight different types in their study. These are bipartite iliac bone plate, accessory SIJ, iliosacral complex, semicircular defect, crescent ilium, isolated synostosis, unfused ossification center and dysmorphic sacrum. The most common type was the iliosacral complex [5]. Ziegeler et al. (2021) reported that the most common joint type was bipartite ilium [8].

Multidisciplinary approaches and detailed evaluation methods are essential for correctly diagnosing and classifying SIJ pathologies [18]. The ability to distinguish similar radiological findings requires the development of existing imaging technologies and the more effective use of these technologies [24]. Integrating new methods and technologies will be crucial to speed up diagnostic processes and increase accuracy in the future. In this process, accurate assessments will be essential in directing patients to appropriate treatment [10,37].

Machine learning-based analytical approaches offer significant advantages in diagnosing SIJ disorders. These algorithms reduce human error and enable accurate interpretation of complex and overlapping imaging features, particularly in early-stage pathological changes. Fu et al. (2024) demonstrated the effectiveness of ML algorithms in distinguishing sacroiliitis from non-inflammatory conditions using CT data, highlighting their clinical applicability [39]. Similarly, Lee et al. (2023) developed an AI model for sacroiliitis grading, achieving up to 100% accuracy across different severity levels [23]. Nicolaes et al. (2024) validated a deep learning model for detecting SIJ inflammation on MRI scans, reporting 70% sensitivity and 81% specificity compared to expert readings, thus demonstrating its feasibility in clinical settings [19]. Kocamış et al. (2025) applied machine learning techniques to predict sex based on SIJ morphometry using 2D and 3D CT images, achieving 88% accuracy with a support vector machine algorithm [20]. However, their study focused solely on anthropometric classification and did not address pathological conditions such as inflammation or degeneration.

In contrast, our study employed machine learning and deep learning algorithms to analyze anatomical features derived from MRI images, classifying SIJ disorders into, control, inflammatory, and degenerative categories. In our findings, SVM (RBF) and XGBoost achieved the highest cross-validated performance, with accuracy and macro-F1 values around 0.95, while RF and LR yielded similarly strong results. The DT model showed comparatively lower performance. The DNN (MLP) model demonstrated stable convergence based on learning curves, although its mean accuracy and macro-F1 were lower than those of the best-performing ML classifiers. Overall, these results suggest that ML-based systems—particularly SVM (RBF) and XGBoost—may support more consistent and objective SIJ assessment and enhance diagnostic decision-making compared to conventional interpretation alone.

## 5. Strengths and Limitations

### 5.1. Strengths

One of the major strengths of this study is the simultaneous analysis of both inflammatory and degenerative SIJ disorders in comparison to a control group, which enables a comprehensive evaluation of structural joint changes across a wide pathological spectrum. The use of standardized MRI protocols and morphometric measurement techniques enhances the reproducibility and objectivity of the findings.

Incorporating multiple supervised machine learning algorithms, including a DNN, allowed for a robust comparison of classification performance, revealing consistent accuracy across models. This multimodal computational approach adds methodological depth and demonstrates the clinical feasibility of AI-driven diagnostic tools in musculoskeletal imaging.

The study also examined anatomical differences related to the disease, providing important insights into the heterogeneity of sacroiliac joint morphology. It demonstrated that joint surface type distribution alone has limited discriminatory value among diagnostic groups. Blinded image evaluations by experienced radiologists, combined with rigorous statistical analysis and cross-validation, further strengthen the methodological rigor of the study.

### 5.2. Limitations

Despite its strengths, the study has some limitations. First, the retrospective and single-center design may limit the generalizability of the findings to other populations or imaging settings.

Second, while MRI provides high-resolution soft tissue contrast, inter-reader variability in joint classification and measurement—although minimized through consensus—may still influence results. Lastly, external validation using an independent dataset was not performed, which would be necessary to confirm the generalizability and clinical applicability of the machine learning models. Additionally, evolutionary neural network (CNN) architectures at the image level (e.g., Dense-Net, ResNet, EfficientNet, VGG) were not evaluated. This is because this study is designed as a feature-based (table) classification using morphological and radiological variables derived from MRI, rather than raw MRI slices. Implementing and comparing CNN models would require a separate image dataset preparation workflow (slice-level labeling/segmentation, fitting, augmentation, and image-based validation), and is therefore outside the scope of the current study. Future studies will expand to raw MRI-based deep learning models and external multi-center validation.

## 6. Conclusions

This study demonstrated that distinct morphological and morphometric alterations occur in the SIJ as a result of inflammatory and degenerative processes, and that these changes can be quantitatively assessed using MRI-based measurements. Narrowing of joint space, presence of erosion and sclerosis, and signal intensity changes were more prevalent in pathological groups, particularly in individuals with inflammatory SIJ disorders.

The integration of these quantitative imaging features into supervised machine learning models (especially SVM (RBF) and XGBoost) yielded high diagnostic performance, with classification accuracies exceeding 95%. Additionally, the deep neural network model achieved comparable outcomes, highlighting its potential to model complex patterns in musculoskeletal imaging.

Overall, this study supports the utility of MRI-based morphometric analysis and machine learning models as objective, reproducible, and clinically relevant tools for distinguishing between inflammatory and degenerative SIJ pathologies. These findings may contribute to improved diagnostic accuracy and facilitate more personalized treatment approaches in patients presenting with sacroiliac joint-related symptoms. Despite anatomical variations across joint types, morphometric and pathological features proved to be more effective discriminators of SIJ pathology.

## Figures and Tables

**Table 1:** Sex and age distribution of participants with SIJ.

Variable	Control	Inflammatory	Degenerative	*p*-Value
Age, mean ± SD (years)	36.8 ± 11.0	41.0 ± 14.0	53.2 ± 8.3	*p* < 0.001
Sex, n (%)				
Male	22 (28.9%)	9 (13.0%)	5 (7.8%)	*p* < 0.002
Female	54 (71.1%)	60 (87.0%)	59 (92.2%)	

**Table 2:** Age, right/left sacroiliac joint space and right/left joint length according to joint problems.

	Control	Inflammatory	Degenerative	Total	*p*
Age	36.80 ± 11.02	40.96 ± 13.95	53.23 ± 8.27	43.21 ± 13.26	0.001
Right SIJ Space (mm)	2.23 ± 0.64	1.83 ± 0.81	2.04 ± 0.57	2.04 ± 0.70	0.002
Left SIJ Space (mm)	2.27 ± 0.59	1.88 ± 0.76	2.13 ± 0.53	2.10 ± 0.65	0.001
Right SIJ A-P length (mm)	34.72 ± 6.79	35.49 ± 5.91	36.52 ± 6.02	35.53 ± 6.29	0.241
Left SIJ A-P length (mm)	34.85 ± 6.62	35.90 ± 6.22	36.40 ± 5.97	35.67 ± 6.30	0.327

**Table 3:** Erosion, sclerosis, inflammation conditions and right/left joint types according to joint problems.

	Control	Inflammatory	Degenerative	Total	*p*
**Sex**					0.002
Male	22	9	5	36	
Female	54	60	59	173	
**Erosion**					0.001
Yes	12	56	63	131	
No	64	13	1	78	
**Sclerosis**					0.001
Yes	8	58	54	120	
No	68	11	10	89	
**Inflammation**					0.001
Active	0	19	1	20	
Chronic	0	18	0	18	
Active + Chronic	0	30	0	30	
Osteitis condensans ilii	0	1	0	1	
No inflammation	76	1	63	140	
**Right Joint Type**					0.852
Normal	50	46	42	138	
Iliosacral Complex	14	13	16	43	
Accessory Joint	7	5	3	15	
Bipartite	4	5	3	12	
Semicircular Defect	1	0	0	1	
**Left Joint Type**					0.935
Normal	50	47	43	140	
Iliosacral Complex	15	13	15	43	
Accessory Joint	7	5	3	15	
Bipartite	3	4	2	9	
Semicircular Defect	1	0	1	2	

**Table 4:** Performance results.

Model	Accuracy	Precision	Recall	F1
Logistic Regression	0.9424 ± 0.0250	0.9445 ± 0.0249	0.9425 ± 0.0250	0.9415 ± 0.0255
SVM-RBF	0.9518 ± 0.0380	0.9518 ± 0.0390	0.9516 ± 0.0386	0.9509 ± 0.0387
Random Forest	0.9472 ± 0.0242	0.9478 ± 0.0242	0.9486 ± 0.0236	0.9464 ± 0.0246
Decision Tree	0.9137 ± 0.0293	0.9147 ± 0.0298	0.9143 ± 0.0312	0.9129 ± 0.0301
XGBoost	0.9518 ± 0.0436	0.9522 ± 0.0440	0.9510 ± 0.0441	0.9506 ± 0.0443
DNN	0.9089 ± 0.0572	0.9132 ± 0.0530	0.9083 ± 0.0583	0.9078 ± 0.0575

## Data Availability

The data that support the findings of this study are available from the corresponding author, upon reasonable request.

## Supplementary Materials

The following supporting information can be downloaded at: https://www.mdpi.com/article/10.3390/diagnostics16050687/s1, Figure S1: Boxplots of numerical variables; Figures S2–S5: Sample MRI images of control, inflammatory, degenerative, and variation cases.

## Abbreviations

The following abbreviations are used in this manuscript:

DT	Decision trees
MRI	Magnetic Resonance Imaging
SIJ	The sacroiliac joint
ML	Machine learning
DL	Deep learning
AI	Artificial intelligence
LR	Logistic regression
SVM	Support vector machine
RF	Random forest
DNN	Deep neural network
