# Supplementary material for: Anatomical Features of the Sacroiliac Joint and Machine Learning-Based Classification of Disease Types
Source: Diagnostics (Basel). 2026 Feb 26;16(5):687. doi: 10.3390/diagnostics16050687 (PMC12984261; doi:10.3390/diagnostics16050687)
Supplement: Supplementary file 1 [file diagnostics-16-00687-s001.zip › diagnostics-4117548-supplementary.pdf]

Supplementary Document

First, data preprocessing and analysis were performed on the data set. Since there was no missing data in the data preprocessing stage, the missing data processing step was not required. Outliers were detected using Z-Score analysis and boxplot graphs. According to the Z-Score method, if a variable deviated more than three units from its standard deviation, it was considered an outlier. In Supplementary Figure S1, boxplot graphs of all numerical variables are presented, and outliers are visually examined. The detected outliers were corrected with the mean values of the relevant variables. This process made the data suitable for analysis without disrupting its general structure.

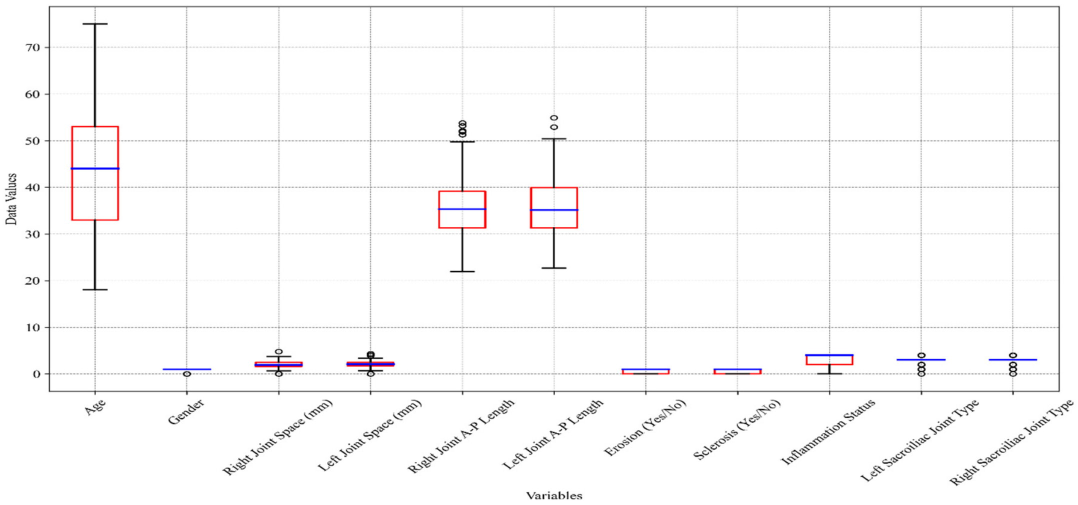

Supplementary Figure S1. Boxplot graphs of all numerical variables.

The patients who were included the study were classified into three groups: those with no pathological findings (control group) (**Figure S2**), the inflammatory group (active sacroiliitis, chronic sacroiliitis) (**Figure S3**), and the degenerative arthritis group (**Figure S4**). The age, gender information of the patients, erosion and sclerosis of the sacroiliac joints were recorded. Additionally, if any variation findings were observed in the sacroiliac joints, they were also recorded (Accessory sacroiliac joint variation, iliosacral complex, and bipartite types were mostly identified) (**Figure S5**).

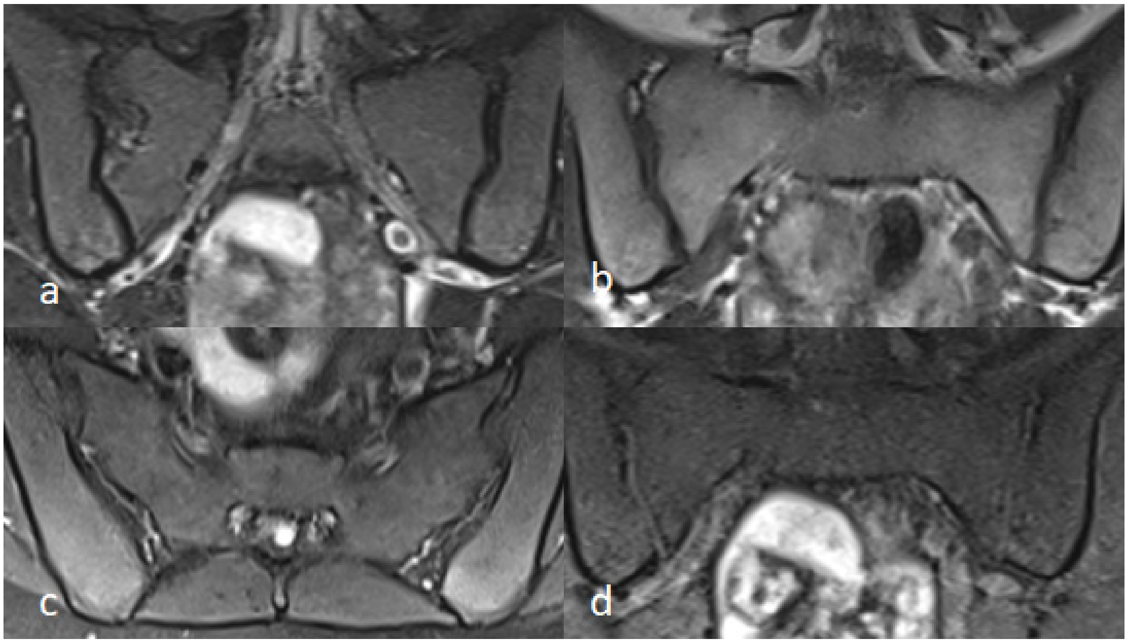

Supplementary Figure S2: Normal sacroiliac joint mri findings of a patient. No findings suggestive of bone marrow edema, synovitis, capsulitis, or enthesitis were observed in the sacroiliac joint regions. No joint space narrowing or significant degeneration has been detected. (a: T2W STIR coronal; b: Coronal T1W image; c: Axial proton density fat suppressed image; d: Coronal T1W fat suppressed image).

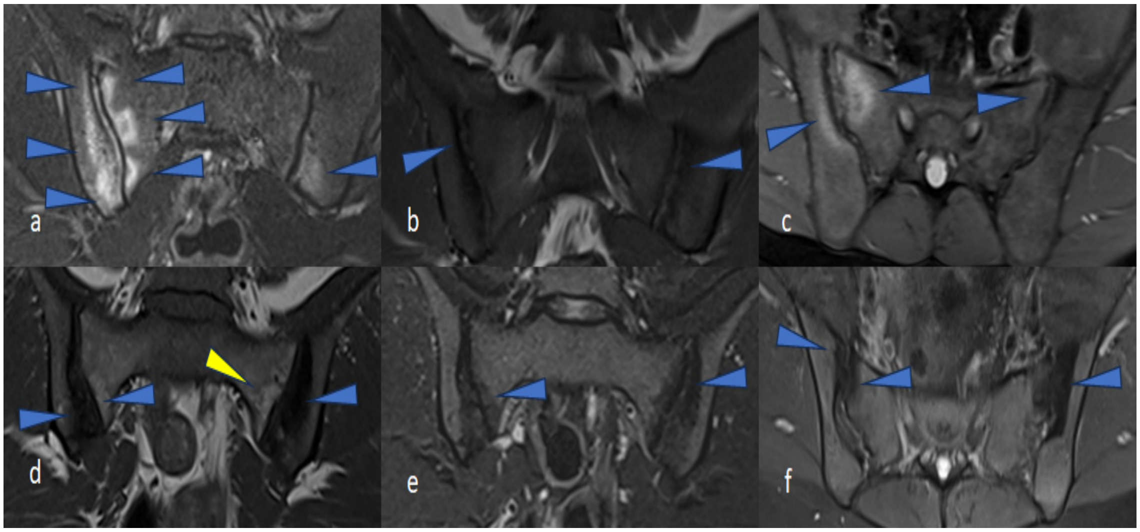

Supplementary Figure S3: In a 23-year-old male patient with ankylosing spondylitis, findings consistent with active sacroiliitis were observed. Bone marrow edema was present in the sacroiliac joint regions bilaterally, more prominent on the right side. On T2 STIR coronal (a) and axial proton density (c) images, hyperintense bone marrow edema was visualized on the joint surfaces. This edema appears hypointense on T1-weighted (T1W) imaging (b). In a 40-year-old female patient (d, e, f) under follow-up for rheumatoid arthritis, degeneration and increased sclerosis of the joint surfaces, joint space narrowing (f), and periarticular fat deposition (a) were observed. Sclerosis appears hypointense in all sequences. Periarticular fat deposition was indicated with a yellow arrowhead. These findings are consistent with chronic sacroiliitis (d: T1W coronal; e: T2W STIR coronal; f: axial proton density fat suppressed image).

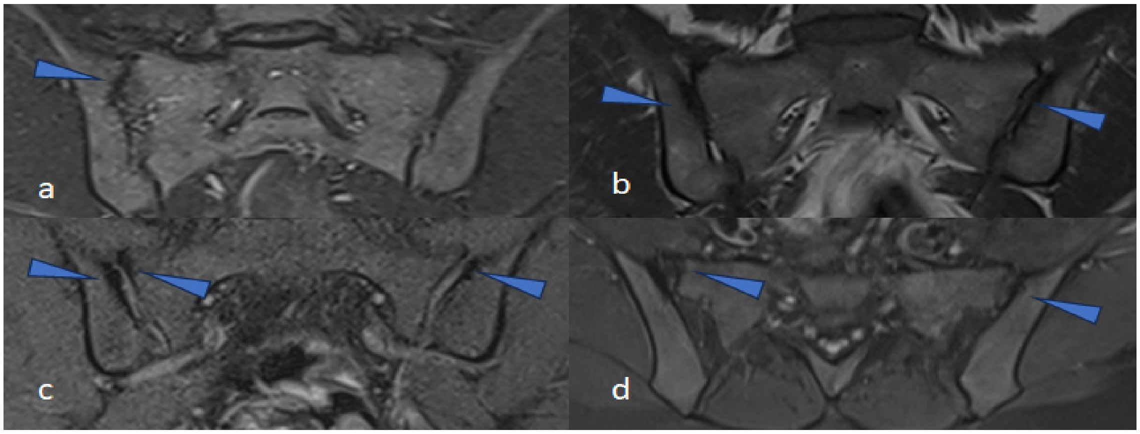

Supplementary Figure S4: In an elderly female patient, joint space narrowing, degenerative cortical irregularities, and increased subchondral sclerosis were observed. This case was reported as degenerative arthritis (a: T2W STIR coronal; b: T1W coronal; c: Axial T1w fat suppressed image; d: Axial proton density fat

suppressed image). Sclerosis appears hypointense in all sequences. Degenerative cortical irregularities in the iliac bone can be observed at the level of the arrowheads in images (a) and (b).

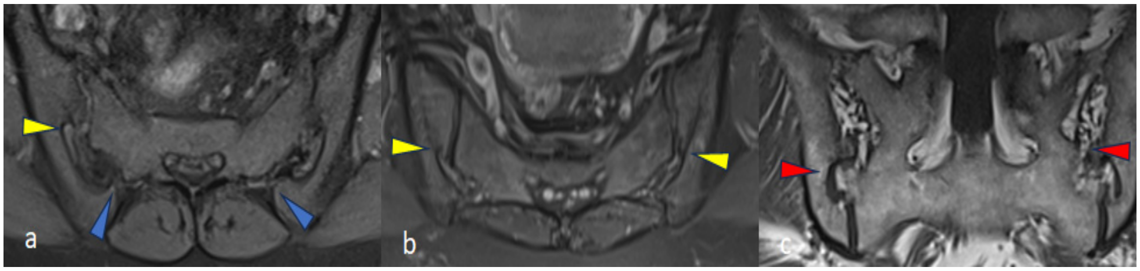

Supplementary Figure S5: In the axial T1 fat-suppressed image (a), bilateral accessory sacroiliac joint variations were observed, indicated by blue arrowheads. In the same patient, a bipartite type was shown in the right sacroiliac joint with a yellow arrow. In the axial T1 fat-suppressed image (b), the bipartite type sacroiliac joint, more prominent on the right side, was indicated by yellow arrowheads on both sides. In the coronal T1-weighted image (c), the appearance consistent with the bilateral iliosacral complex was shown with red arrowheads.
